# Synthesis and Biological activity of 4-(4,6-Disubstituted-pyrimidin-2-yloxy)phenoxy Acetates

**DOI:** 10.3390/molecules15021074

**Published:** 2010-02-23

**Authors:** Lin Jiang, Hao Wang, Maorong Wang, Xinhuan Teng

**Affiliations:** 1 College of Chemistry and Material Science, Shandong Agricultural University, Taian 271018, China; E-Mails: hw@sdau.edu.cn (H.W.), maorongwang@163.com (M.R.W.); 2 College of Plant Protection, Shandong Agricultural University, Taian 271018, China; E-Mail: tengxinhuan_0505@163.com (X.H.T.)

**Keywords:** pyrimidine, phenoxyacetate, synthesis, herbicidal activity

## Abstract

Ten novel 4-(4,6-dimethoxypyrimidin-2-yloxy)phenoxy acetates and 4-(4,6-dimethylpyrimidin-2-yloxy)phenoxy acetates were synthesized with hydroquinone, 2-methylsulfonyl-4,6-disubstituted-pyrimidine and chloroacetic ester as starting materials. The products were characterized by IR, ^1^H-NMR, MS spectra and elemental analyses. Preliminary bioassay indicates that the target compounds possess high herbicidal activity against monocotyledonous plants such as *Digitaria sanguinalis* L. at concentrations of 100 mg/L and 50 mg/L.

## 1. Introduction

Aryloxy-phenoxy propionates are an important class of herbicides due to their high efficiency, broad spectrum, low toxicity and good selectivity. They act by blocking the biosynthesis of fatty acids by inhibiting acetyl-coenzyme A carboxylase [1,2,3]. Since the first herbicide of this series, diclofop-methyl, was synthesized in 1972, more than twenty aryloxy-phenoxy propionate herbicides such as fluazifop-butyl, heloxyfop-methyl, quizalofop-ethyl and cyhalofop-butyl have been developed [4], and are widely used to control gramineous weeds. In addition, some aryloxy-phenoxy acetates exhibit good herbicidal activity. For example, two substituted pyrazolo[3,4-d] pyrimidin-4-yloxy phenoxy acetates display considerable activities [5], with 100% inhibition against the root growth of *Brassica napus* L. at a concentration of 100 mg/L, and 98.1% and 100% against the root growth of *Echinochloa crusgalli* L. at the same concentration, respectively. 

On the other hand, a lot of compounds containing 4,6-dimethoxypyrimidin-2-yl or 4,6-dimethylpyrimidin-2-yl moieties display excellent herbicidal activity. Most sulfonylurea herbicides and all pyrimidinylbenzoate herbicides (two series of super-efficient herbicides) [6,7,8,9,10], such as nicofulfuron, amidosulfuron, halopyrazosulfuron, ethoxysulfuron, pyriminobac-methyl and pyriftalid, possess 4,6-dimethoxypyrimidin-2-yl groups, while sulfometuron-methyl, a kind of sulfonylurea, contains a 4,6-dimethylpyrimidin-2-yl group, which suggests that the two disubstituted-pyrimidin-2-yl groups possess high biological activity [11]. In order to seek efficient herbicidal compounds, herein we introduced 4,6-dimethoxypyrimidin-2-yl or 4,6-dimethylpyrimidin-2-yl into aryloxy-phenoxy acetates by the sub-structure link route, synthesizing ten novel 4-(4,6-dimethoxypyrimidin-2-yloxy)phenoxy acetates and 4-(4,6-dimethylpyrimidin-2-yloxy)phenoxy acetates. 

## 2. Results and Discussion

### 2.1. Synthesis

**Scheme 1:**
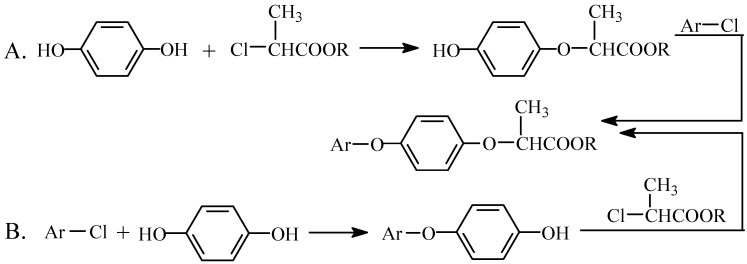
Synthetic pathways of aryloxy-phenoxy propionates.

There are two pathways (route A and route B, Scheme 1) in the synthesis of aryloxy-phenoxy propionate herbicides [12]. In route A, hydroquinone reacts with 2-chloropropionates to yield 4-hydroxyphenoxy propionates which react with aryl chlorides to give the target compounds. In route B, hydroquinone firstly reacts with an aryl chloride to give a 4-hydroxyphenyl aryl ether which reacts with 2-chloropropionates to give the target compound. 

**Scheme 2:**
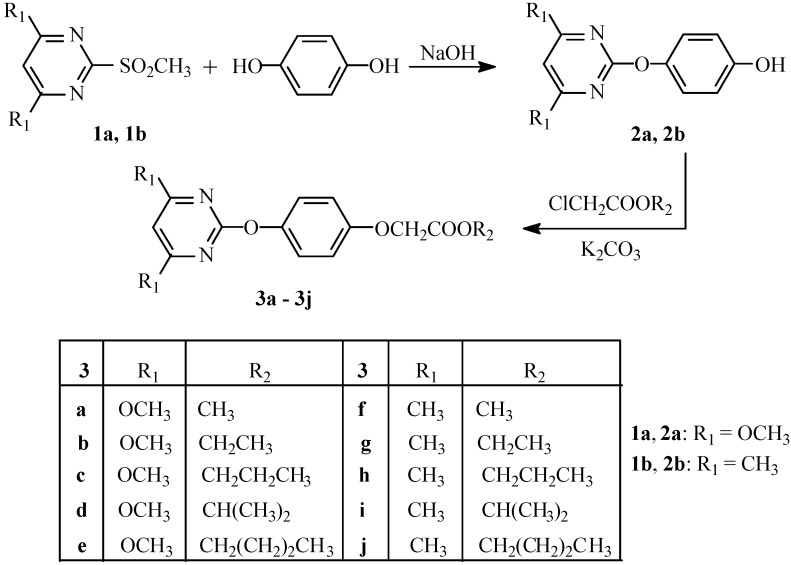
Synthetic route of target compounds **3a–3j**.

Considering the lower yield of 4-hydroxyphenoxy propionate in the reaction of hydroquinone with 2-chloropropionate in route A, we choose route B as the synthetic strategy in our work. Moreover, we used 2-methylsulfonyl-4,6-disubstitutedpyrimidines instead of 2-chloro-4,6-disubstitutedpyrimidines to produce 2-(4-hydroxyphenoxy)-4,6-disubstitutedpyrimidines **2a, 2b** owing to their higher activity [13]. The reaction of hydroquinone with 2-methylsulfonyl-4,6-dimethoxylpyrimidine in tetrahydrofuran or *N*,*N-*dimethylformamide at 70–80 ºC [14] gives **2a** in very low yield and low purity. Hence, depending on the preparation method of 2-(4-hydroxyphenoxy)-6-chlorobenzoxazole [15], we used sodium hydroxide as a base, and benzyltriethylammonium chloride as a phase transfer catalyst, thus, refluxing the hydroquinone with 2-methylsulfonyl-4,6-dimethoxylpyrimidine for 3.5 h with stirring in a mixed solvent of toluene and water affords compound **2a** with a satisfactory yield. The reaction of **2a** (or **2b**) with the appropriate chloroacetates in acetonitrile in the presence of potassium carbonate gives compounds **3a**–**3j **(Scheme 2).

Both spectral and elemental analyses data of the prepared compounds **3a**–**3j** were all in agreement with the suggested molecular structures (see Experimental). Their IR spectra exhibited absorption bands assignable to C=O stretching vibrations and characteristic bands near υ 1208–1193 cm^−1^and 1084–1062 cm^−1^, corresponding to the C-O-C linkages. In addition, the ^1^H-NMR spectra of **3a**–**3j** showed characteristic singlet signals at δ 4.59–4.64, 3.82–3.83, and 2.39 ppm, assignable to the methylene, methoxy and methyl protons, respectively, and singlet signals at δ 5.76 ppm or at δ 6.74–6.75 ppm corresponding to the dimethoxy- or dimethylpyrimidine ring protons (5-H), in addition to the expected aromatic protons appear as double doublet signals at 6.91–6.93 and 7.12–7.14 ppm, respectively.

### 2.2. Biological Activity

The herbicidal activities are summarized in Table 1. In all tested compounds, the rates of inhibition of *Brassica napus* L. root growth are 15.9%–58.4%, 13.1%–36.4% and 9.2%–22.9% at concentrations of 100 mg/L, 50 mg/L and 10 mg/L, respectively, which means that the synthesized compounds display low herbicidal activities against this plant. However, the rates of inhibition of *Digitaria sanguinalis* L. root growth are all 100% at a concentration of 100 mg/L, 95.2%–98.5% at 50 mg/L, and 32.5%–44.7% at 10 mg/L. The results demonstrate that the target compounds exhibit excellent herbicidal activities against this kind of weed at 100 mg/L and 50 mg/L, but their activities are not satisfactory at a lower concentration (10 mg/L). 

**Table 1:** Herbicidal activities of compounds **3a**–**3j**.

Compd.	Inhibitory rate (%)					
	*Brassica napus* L.			*Echinochloa crusgalli* L.		
	100 mg/L 50 mg/L 10 mg/L			100 mg/L 50 mg/L 10 mg/L		
**3a**	39.8	34.4	22.9	100	98.5	42.8
**3b**	41.1	31.0	13.4	100	96.2	39.9
**3c**	15.9	13.1	10.1	100	95.2	32.5
**3d**	25.1	21.0	14.6	100	96.5	32.7
**3e**	28.3	13.4	9.5	100	96.9	41.8
**3f**	44.7	26.7	11.9	100	97.3	44.7
**3g**	58.4	32.7	14.2	100	98.4	38.5
**3h**	54.5	33.9	12.1	100	97.5	36.9
**3i**	26.4	14.3	9.2	100	95.1	35.0
**3j**	50.2	34.2	21.5	100	97.6	42.6
quizalofop-P-ethyl	51.2	39.2	33.8	100	95.3	63.5

## 3. Experimental

### 3.1. General

Melting points were measured on an X-5 microscopic melting-point apparatus and uncorrected. IR spectra were recorded in KBr pellets on a Shimadzu IR-440 infrared spectrophotometer. ^1^H-NMR spectra were registered on an Inova-600 spectrometer (in CDCl_3_ solvent, TMS as internal standard). Mass spectra were recorded on an Agilent 1100 LC-MS spectrometer (APCI source). Elemental analyses were performed with a Vario EL III Elemental Analyzer. 

### 3.2. General method for the synthesis of 2-(4-hydroxyphenoxy)-4,6-disubstitutedpyrimidines ***2a**, **2b***

To a solution of sodium hydroxide (1.20 g, 30.0 mmol) and benzyltriethylammonium chloride (0.10 g, 0.4 mmol) in water (15 mL) were added hydroquinone (2.98 g, 27.0 mmol) and toluene (7 mL). The resulting mixture was heated to 60 ºC while stirring under nitrogen, then 2-methylsulfonyl-4,6-disubstitutedpyrimidine **1a** or **1b** (18.0 mmol) in dichloromethane (8 mL) was added dropwise in 1 h. The reaction mixture was further refluxed with stirring for 3 h, then toluene and dichloromethane were evaporated in a rotary evaporator. Sodium hydroxide (5%, 30 mL) was added to the residue, and extracted with ethyl acetate (10 mL × 2). The aqueous layer was acidified with concentrated hydrochloric acid to pH4–5. The precipitated solid was filtered off, washed with water, dried and crystallized from a mixed solvent of ethanol and water (V:V = 1:2) to give **2a**, **2b** respectively.

*2-(4-Hydroxyphenoxy)-4,6-dimethoxypyrimidine* (**2a**): Yield: 86.5%; m.p. 120.1-121.8 ºC; ^1^H-NMR δ: 3.84 (s, 6H, CH_3_), 5.76 (s, 1H, Pyrim-H), 5.79 (s, 1H, OH), 6.82 (d, 2H, *J* = 8.4 Hz, Ph-H), 7.03 (d, 2H, *J* = 8.4 Hz, Ph-H) ppm; IR (KBr): 3453, 1620, 1573, 1513, 1454, 812 cm^−1^; MS (APCI) m/z: 249.9 (M + 2H)^+^; Anal. for C_12_H_12_N_2_O_4_ (%): C 58.06, H 4.87, N 11.28; found C 58.31, H 4.74, N 11.41. 

*2-(4-Hydroxyphenoxy)-4,6-dimethylpyrimidine* (**2b**): Yield: 90.6%, m.p. 195.8-197.2 ºC; ^1^H-NMR δ: 2.41 (s, 6H, CH_3_), 6.76 (s, 1H, Pyrim-H), 6.78 (d, 2H, *J* = 8.4 Hz, Ph-H), 6.98 (d, 2H, *J* = 8.4 Hz, Ph-H), 7.45 (s, 1H, OH) ppm; IR (KBr): 3448, 1637, 1560, 1509, 1458, 820 cm^−^^1^; MS (APCI) m/z: 218.0 (M + 2H)^+^; Anal. for C_12_H_12_N_2_O_2_ (%): C 66.65, H 6.66, N 12.95; found C 66.69, H 6.21, N 12.91.

### 3.3. General method for the preparation of 4-(4,6-disubstitutedpyrimidin-2-yloxy)phenoxy acetates ***3a–3j***

To a mixture of 2-(4-hydroxyphenoxy)-4,6-disubstitutedpyrimidine **2a** or **2b** (5.0 mmol) and anhydrous potassium carbonate (0.97 g, 7.0 mmol) in acetonitrile (10 mL), was added the appropriate chloroacetic ester (6.0 mmol) while stirring. The reaction mixture was refluxed with stirring for 7 h. The solvent was removed in a rotary evaporator, then, the residue was poured into ice-water (20 mL), and leave aside for 1 h to solidify. The solid so formed was filtered off, washed with ice-water, dried and crystallized from a mixed solvent of petroleum ether and ethyl acetate (V: V = 5:1–2) to afford **3a–3j**. 

*Methyl 4-(4,6-dimethoxypyrimidin-2-yloxy)phenoxy acetate* (**3a**): Yield: 81.2%; m.p. 77.5–79.0 ºC; ^1^H-NMR δ: 3.82 (s, 3H, COOCH_3_), 3.83 (s, 6H, Pyrim-OCH_3_), 4.64 (s, 2H, OCH_2_), 5.76 (s, 1H, Pyrim-H), 6.92 (d, 2H, *J* = 9.0 Hz, Ph-H), 7.14 (d, 2H, *J* = 9.0 Hz, Ph-H) ppm; IR (KBr): 1754, 1205, 1083, 820 cm^−^^1^; MS (APCI) m/z: 321.9 (M + 2H)^+^; Anal. for C_15_H_16_N_2_O_6_ (%): C 56.25, H 5.04, N 8.74; found C 56.51, H 4.99, N 8.79.

*Ethyl 4-(4,6-dimethoxypyrimidin-2-yloxy)phenoxy acetate* (**3b**): Yield: 78.5%; m.p. 75.2–76.1 ºC; ^1^H-NMR δ: 1.30 (t, 3H, *J* = 7.2 Hz, CH_2_-CH_3_), 3.83 (s, 6H, Pyrim-OCH_3_), 4.28 (q, 2H, *J* = 7.2 Hz, COOCH_2_), 4.62 (s, 2H, OCH_2_), 5.76 (s, 1H, Pyrim-H), 6.92 (d, 2H, *J* = 9.0 Hz, Ph-H), 7.14 (d, 2H, *J* = 9.0 Hz, Ph-H) ppm; IR (KBr): 1758, 1205, 1068, 825 cm^−^^1^; MS (APCI) m/z: 335.9 (M + 2H)^+^; Anal. for C_16_H_18_N_2_O_6_ (%): C 57.48, H 5.43, N 8.38; found C 57.75, H 5.35, N 8.25.

*Propyl 4-(4,6-dimethoxypyrimidin-2-yloxy)phenoxy acetate* (**3c**): Yield: 75.6%; m.p. 82.8–84.3 ºC; ^1^H-NMR δ: 0.93 (t, 3H, *J* = 7.2 Hz, CH_2_-CH_3_), 1.69 (m, 2H, CH_2_-CH_3_), 3.83 (s, 6H, Pyrim-OCH_3_), 4.18 (t, *J* = 6.6 Hz, 2H, COOCH_2_), 4.63 (s, 2H, OCH_2_), 5.76 (s, 1H, Pyrim-H), 6.92 (d, 2H, *J* = 9.0 Hz, Ph-H), 7.14 (d, 2H, *J* = 9.0 Hz, Ph-H) ppm; IR (KBr) 1759, 1206, 1066, 829 cm^−^^1^; MS (APCI) m/z: 350.0 (M + 2H)^+^; Anal. for C_17_H_20_N_2_O_6_ (%): C 58.61, H 5.79, N 8.04; found C 58.71, H 5.72, N 8.05.

*Isopropyl 4-(4,6-dimethoxypyrimidin-2-yloxy)phenoxy acetate* (**3d**): Yield: 80.9%; m.p. 79.8–81.5 ºC; ^1^H-NMR δ: 1.28 (d, 6H, *J* = 6.6 Hz, CH-(CH_3_)_2_), 3.82 (s, 6H, Pyrim-OCH_3_), 4.59 (s, 2H, OCH_2_), 5.15 (m, 1H, COOCH), 5.76 (s, 1H, Pyrim-H), 6.91 (d, 2H, *J* = 9.0 Hz, Ph-H), 7.12 (d, 2H, *J* = 9.0 Hz, Ph-H) ppm; IR (KBr): 1760, 1213, 1062, 815 cm^−^^1^; MS (APCI) m/z: 349.9 (M + 2H)^+^; Anal. for C_17_H_20_N_2_O_6_ (%): C 58.61, H 5.79, N 8.04; found C 58.65, H 5.71, N 8.02.

*Butyl 4-(4,6-dimethoxypyrimidin-2-yloxy)phenoxy acetate* (**3e**): Yield: 71.3%; m.p. 70.1–71.3 ºC; ^1^H-NMR δ: 0.93 (t, 3H, *J* = 7.2 Hz, CH_2_-CH_3_), 1.36 (m, 2H, CH_2_-CH_3_), 1.64 (m, 2H, CH_2_–C_2_H_5_), 3.83 (s, 6H, Pyrim-OCH_3_), 4.22 (t, 2H, *J* = 6.6 Hz, COOCH_2_), 4.63 (s, 2H, OCH_2_), 5.76 (s, 1H, Pyrim-H), 6.91 (d, 2H, *J* = 9.0 Hz, Ph-H), 7.14 (d, 2H, *J* = 9.0 Hz, Ph-H) ppm; IR (KBr): 1763, 1208, 1082, 809 cm^−^^1^; MS (APCI) m/z: 363.9 (M + 2H)^+^; Anal. for C_18_H_22_N_2_O_6_ (%): C 59.66, H 6.12, N 7.73; found C 59.49, H 6.08, N 7.77.

*Methyl 4-(4,6-dimethylpyrimidin-2-yloxy)phenoxy acetate* (**3f**): Yield: 80.1%; m.p. 114.5–116.0 ºC; ^1^H-NMR δ: 2.39 (s, 6H, Pyrim-CH_3_), 3.82 (s, 3H, COOCH_3_), 4.64 (s, 2H, OCH_2_), 6.75 (s, 1H, Pyrim-H), 6.92 (d, 2H, *J* = 9.0 Hz, Ph-H), 7.13 (d, 2H, *J* = 9.0 Hz, Ph-H) ppm; IR (KBr): 1753, 1200, 1082, 833 cm^−^^1^. MS (APCI) m/z: 289.9 (M + 2H)^+^; Anal. for C_15_H_16_N_2_O_4_ (%): C 62.53, H 5.59, N 9.71; found C 62.86, H 5.55, N 9.70.

*Ethyl 4-(4,6-dimethylpyrimidin-2-yloxy)phenoxy acetate* (**3g**): Yield: 77.2%; m.p. 90.2–92.1 ºC; ^1^H-NMR δ: 1.31 (t, 3H, *J* = 7.2 Hz, CH_2_-CH_3_), 2.39 (s, 6H, Pyrim-CH_3_), 4.28 (q, 2H, *J* = 7.2 Hz, COOCH_2_), 4.62 (s, 2H, OCH_2_), 6.74 (s, 1H, Pyrim-H), 6.92(d, 2H, *J* = 9.0 Hz, Ph-H), 7.13(d, 2H, *J* = 9.0 Hz, Ph-H) ppm; IR (KBr): 1758, 1197, 1078, 845 cm^−^^1^; MS (APCI) m/z: 303.9 (M + 2H)^+^; Anal. for C_16_H_18_N_2_O_4_ (%): C 63.62, H 6.01, N 9.26; found C 64.03, H 5.98, N 9.33.

*Propyl 4-(4,6-dimethylpyrimidin-2-yloxy)phenoxy acetate* (**3h**): Yield: 75.0%; m.p. 95.4–97.3 ºC; ^1^H-NMR δ: 0.93 (t, 3H, *J* = 7.2 Hz, CH_2_-CH_3_), 1.69 (m, 2H, CH_2_-CH_3_), 2.39 (s, 6H, Pyrim-CH_3_); 4.18 (t, *J* = 6.6 Hz, 2H, COOCH_2_), 4.64 (s, 2H, OCH_2_), 6.74 (s, 1H, Pyrim-H), 6.93 (d, 2H, *J* = 9.0 Hz, Ph-H), 7.13(d, 2H, *J* = 9.0 Hz, Ph-H) ppm; IR (KBr): 1760, 1198, 1078, 849 cm^−^^1^; MS (APCI) m/z: 317.9 (M + 2H)^+^; Anal. for C_17_H_20_N_2_O_4_ (%): C 64.54, H 6.37, N 8.85; found C 64.84, H 6.28, N 8.76.

*Isopropyl 4-(4,6-dimethylpyrimidin-2-yloxy)phenoxy acetate* (**3i**): Yield: 80.9%; m.p. 90.5–92.2 ºC; ^1^H-NMR δ: 1.28 (d, 6H, *J* = 6.6 Hz, CH-(CH_3_)_2_), 2.39 (s, 6H, Pyrim-CH_3_), 4.59 (s, 2H, OCH_2_), 5.15 (m, 1H, COOCH), 6.74 (s, 1H, Pyrim-H), 6.92 (d, 2H, *J* = 9.0 Hz, Ph-H), 7.12 (d, 2H, *J* = 9.0 Hz, Ph-H) ppm; IR (KBr): 1721, 1204, 1073, 854 cm^−^^1^; MS (APCI) m/z: 317.9 (M + 2H)^+^; Anal. for C_17_H_20_N_2_O_4_ (%): C 64.54, H 6.37, N 8.85; found C 64.76, H 6.29, N 8.68.

*Butyl 4-(4,6-dimethylpyrimidin-2-yloxy)phenoxy acetate* (**3j**): Yield: 71.3%; m.p. 34.2–36.0 ºC; ^1^H-NMR δ: 0.92 (t, 3H, *J* = 7.2 Hz, CH_2_-CH_3_), 1.37 (m, 2H, CH_2_-CH_3_), 1.65 (m, 2H, CH_2_–C_2_H_5_), 2.39 (s, 6H, Pyrim-CH_3_), 4.22 (t, 2H, *J* = 6.6 Hz, COOCH_2_), 4.63 (s, 2H, OCH_2_), 6.74 (s, 1H, Pyrim-H), 6.92 (d, 2H, *J* = 9.0 Hz, Ph-H), 7.13(d, 2H, *J* = 9.0 Hz, Ph-H) ppm; IR (KBr): 1752, 1193, 1084, 830 cm^−^^1^. MS (APCI) m/z: 331.9 (M + 2H)^+^; Anal. for C_18_H_22_N_2_O_4_ (%): C 65.44, H 6.71, N 8.48; found C 65.14, H 6.74, N 8.21.

### 3.4. Biological activity

The herbicidal activities of the target compounds were determined using *Brassica napus* L. and *Digitaria sanguinalis* L. as samples of dicotyledonous and monocotyledonous plants, respectively [16,17]. Emulsions of the tested compounds were prepared by dissolving them in *N*,*N-*dimethylformamide (100 μL) with the addition of Tween 20 (2 μL), and then diluting with distilled water. The germinated seeds were placed on two filter papers in a 9-cm Petri plate, to which 5 mL of tested solution was added in advance. Usually, 15 seeds were used on each plate. The plates were placed in a dark room and allowed to germinate for 65 h at 28 (±1) ºC. The lengths of 10 seed roots selected from each plate were measured and the means were calculated. Moreover, quizalofop-P-ethyl, a commercial aryloxy-phenoxy propionate herbicide and the emulsion which does not contain tested compounds were used as control and blank respectively. For all of the bioassay tests, each treatment was repeated three times. The inhibitory rate was calculated relative to the blank. The bioassay results are shown in Table 1.

## 4. Conclusions

A new method of preparing 2-(4-hydroxyphenyloxy)-4,6-disustitutedpyrimidine has been developed with the advantages of a shorter reaction time, fewer side-products and higher yields. Ten novel 4-(4,6-dimethoxypyrimidin-2-yloxy)phenoxy acetate and 4-(4,6-dimethylpyrimidin-2-yloxy)phenoxy acetate compounds have been synthesized, and characterized by IR, ^1^H-NMR, MS spectra and elemental analyses. Preliminary bioassay indicates that the target compounds exhibit high herbicidal activity against monocotyledonous plant such as *Digitaria sanguinalis* L. at concentrations of 100 mg/L and 50 mg/L.
